# Imaging of Molecular and Developmental Responses to Abiotic Stresses in Reproductive Tissues

**DOI:** 10.1111/ppl.70759

**Published:** 2026-02-02

**Authors:** Hana Daryanavard, Teresa Paraiso, María Cielo Pasten, Bianca Maria Orlando Marchesano, Marta Adelina Mendes, Hélène S. Robert, Francesca Resentini, Joëlle K. Mühlemann

**Affiliations:** ^1^ Leuven Plant Institute KU Leuven Leuven Belgium; ^2^ Department of Biosystems KU Leuven Leuven Belgium; ^3^ Mendel Center for Plant Genomics and Proteomics, Central European Institute of Technology Masaryk University Brno Czech Republic; ^4^ Laboratory of Functional Genomics and Proteomics, National Centre for Biomolecular Research, Faculty of Science Masaryk University Brno Czech Republic; ^5^ Centro de Recursos Naturales Renovables de la Zona Semiárida (CERZOS), CONICET Bahía Blanca Buenos Aires Argentina; ^6^ Department of Biosciences University of Milan Milano Italy

**Keywords:** abiotic stress responses, calcium, imaging approaches, plant reproduction, ROS

## Abstract

Abiotic stresses, such as drought, salinity, and extreme temperatures, have profound effects on plant reproduction, often leading to reduced fertility and yield. Reproduction in plants involves complex interactions between diverse cells, necessitating spatiotemporal resolution to understand how stress impacts each component of this intricate system. Imaging techniques have emerged as indispensable tools for uncovering the cellular and molecular responses of reproductive tissues to abiotic stresses in Arabidopsis and crops. Advanced methods, including fluorescence‐based dyes and genetically encoded biosensors, have enabled the visualization of key stress‐associated molecules such as reactive oxygen species and calcium ions. These approaches reveal the dynamic and localized nature of stress responses. Additionally, state‐of‐the‐art imaging technologies, including light‐sheet microscopy, structured illumination (e.g., Apotome), high‐content confocal microscopy, micro‐computed tomography, and custom heated‐stage setups, provide varying levels of spatial and temporal resolution to study stress‐induced changes in tissue morphology and development. Complementary techniques like sectioning and staining continue to yield critical insights into the anatomical and developmental alterations under stress conditions. This review integrates findings from these methodologies, highlighting their contributions to our understanding of abiotic stress responses in male and female reproductive tissues. Furthermore, we identify technological advancements needed to enable real‐time, (sub)cellular‐level imaging of stress responses. Finally, we compile a list of promoter‐based identity markers specific to reproductive tissues across different crop species, offering a resource for targeted genetic studies. By bridging current imaging techniques with biological insights and technological gaps, this work aims to advance the field of plant stress biology and reproductive resilience.

## 1 Plant Reproduction Is Highly Sensitive to Abiotic Stresses

Plant reproduction underpins agricultural productivity and food security, while also ensuring the evolutionary success of plant species in natural ecosystems. At the same time, reproductive tissues are highly sensitive to abiotic stresses linked to climate change, such as extreme temperatures, varying water availability, and increased soil salinity (RECROP COST 2025). This sensitivity to abiotic stresses is a major cause of reduced crop yields in the face of climate change and a threat to plant populations in natural ecosystems. Therefore, understanding how climate change affects reproductive tissues is crucial, especially for the development of climate‐resilient crops.

Abiotic stresses can disrupt each stage of reproduction, from male and female gametophyte development to the progamic phase, fertilization, and seed development (RECROP COST 2025). The different reproductive tissues have their own developmental timelines, ‘omic fingerprints, and physiological dynamics. As a result, they often display tissue‐specific sensitivities and unique morphological, molecular, and physiological responses when exposed to abiotic stresses. Imaging approaches provide powerful means to uncover these tissue‐specific stress responses in real time and/or at high spatial resolution. This article not only describes the responses of diverse reproductive tissues to abiotic stresses but also highlights the imaging approaches (Table 1) that can be used to monitor developmental, physiological, and molecular perturbations in reproductive tissues under abiotic stress.

## 2 Visualizing Developmental Defects in Plant Reproduction Under Abiotic Stress

Successful plant reproduction involves three major events: generation of male and female gametophytes, fertilization, and seed development. The male gametophyte, known as pollen, develops in the anthers of the flower. Its development starts with microsporogenesis, during which a microspore mother cell undergoes meiosis to generate tetrads of haploid microspores (Hafidh and Honys 2021). These then go through mitosis to form mature pollen grains, which carry two sperm cells (Gómez et al. 2015), in a process termed microgametogenesis. The female gametophyte, also known as the embryo sac, develops within the ovules. Analogously to pollen development, ovule development has been divided into two main phases: megasporogenesis, involving the formation of functional megaspores by meiosis; and megagametogenesis, during which the mature embryo sac develops. The pattern of embryo sac formation during sexual reproduction varies among species, and it has been classified into different types, the most frequent one being the polygonum‐type embryo sac, found in over 70% of flowering plants (Drews and Koltunow 2011; Schneitz et al. 1995; Willemse and van Went 1984). The mature embryo sac contains 6 haploid cells: the egg cell, two synergid cells, three antipodal cells, and one diploid cell known as the central cell. Upon pollination, mature pollen lands on the stigma of the pistil and germinates to form a tube that penetrates the stigma and elongates down the style to reach the ovule (Hafidh and Honys 2021). The pollen tube then bursts to release the sperm cells, upon receptive synergid cell degeneration, into the embryo sac (Mendes et al. 2015). Double fertilization occurs when one sperm cell fuses with the egg to form a zygote, and the other sperm cell fuses with the central cell to generate the endosperm (Hater et al. 2020). This fertilization event triggers seed development (Figueiredo and Sharma 2025; Mizzotti et al. 2012). The mature seed consists of three interconnected tissues: a maternal, sporophytic seed coat that protects the triploid endosperm and the diploid zygotic embryo. Seed development is divided into two phases: growth and morphogenesis, during which the embryo acquires the morphological features of the future seedling, followed by seed maturation and dormancy. This intricate developmental sequence is highly sensitive to diverse abiotic stresses, which can disrupt multiple stages and lead to reproductive defects. In this section, we highlight imaging approaches used to visualize and characterize these stress‐induced defects.

### 2.1 Pollen Development and Tube Growth

**FIGURE 1:**
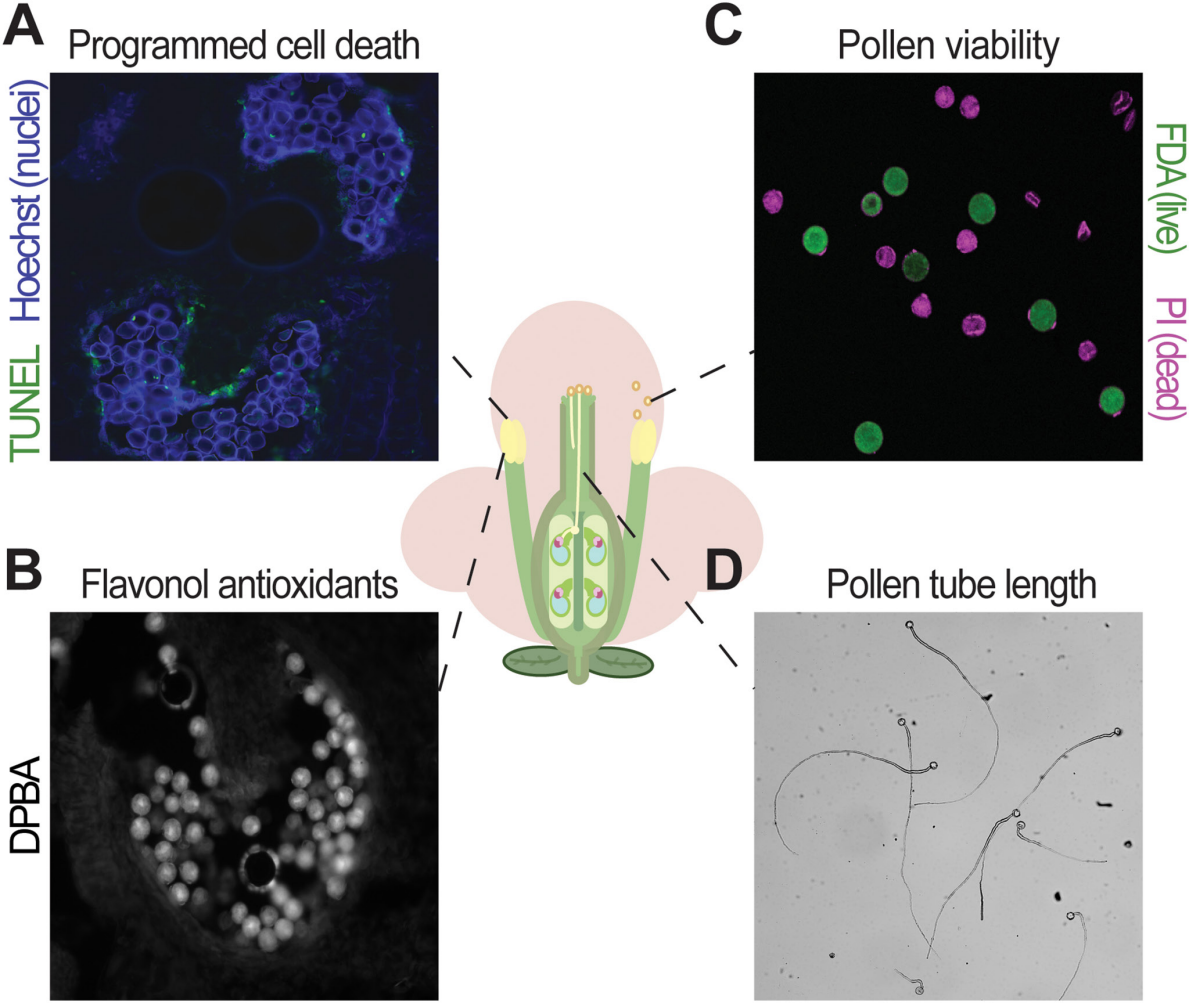
Imaging methodologies to monitor pollen development. The schematic of an angiosperm flower in the center shows the anther tissue and pollen developmental stages where the highlighted imaging approaches were used. (A) Visualization of programmed cell death in a cross section of a tomato anther using the TUNEL assay (epifluorescence microscopy). (B) Diphenylboric acid 2‐aminoethylester (DPBA) staining of a tomato anther cross‐section to localize and quantify flavonol antioxidants (epifluorescence microscopy). (C) Pollen viability analysis with fluorescein diacetate (FDA, live pollen) and propidium iodide (PI, dead pollen) in tomato (imaged using confocal laser scanning microscopy, CLSM). (D) Pollen tubes of tomato grown in vitro and imaged using bright‐field microscopy.

Abiotic stresses, such as heat, cold, and drought, affect male reproductive development on various levels, from generating oxidative stress to causing transcriptional remodeling and morphological changes (Zhang et al. 2021). Proper pollen development depends on a sporophytic cell layer termed tapetum. Tapetal cells surround the developing pollen and support it by providing crucial nutrients, metabolites, enzymes, and cell wall material (Gómez et al. 2015). Coordination between tapetum and pollen development is essential for the production of viable pollen. Indeed, the tapetum undergoes programmed cell death (PCD), whose timing and progression is a highly intricate process that is in part regulated by reactive oxygen species (ROS) (see Section 2.2.) (Xie et al. 2014; Ye et al. 2021). Disruptions in this process, in turn, disrupt the process of microspore development, resulting in non‐viable pollen. PCD can be measured using the TdT‐mediated dUTP nick‐end labeling (TUNEL) technique, which involves fluorescent tagging of DNA double‐strand breaks (a hallmark of apoptosis). The TUNEL technique allows for in situ visualization of tapetal PCD when applied to transverse sections of anther tissue (Figure 1A). Several studies have focused on changes in the tapetum in response to abiotic stresses. Sectioned rice anthers subjected to the TUNEL assay showed higher signal intensity in the tapetum under heat stress compared to non‐stressed conditions (Feng et al. 2018; Zhao, Guan, et al. 2023). Higher TUNEL signal in the tapetum of rice anther sections in response to heat stress also coincides with higher ROS levels, which are a hallmark of abiotic stresses (Zhao, Guan, et al. 2023). While high temperature stress exacerbates tapetal cell death, in watermelon, the application of cold stress (15°C) has been shown to inhibit tapetal PCD based on the TUNEL assay (Lyu et al. 2019).

In parallel to its effects on tapetum degeneration, abiotic stress is known to greatly affect the abundance and viability of pollen grains. Therefore, measures of pollen viability are widely used to assess abiotic stress responses and tolerance. Non‐fluorescent dyes such as iodine and potassium iodide solution (I_2_‐KI) can be used to stain pollen and assess viability, where black‐stained pollen is considered viable. I_2_‐KI staining of cotton pollen grains showed lower pollen viability under individual and combined drought and heat stress, with a larger decrease in viability observed under combined stress (Zhang et al. 2023). Rice pollen grains stained with I_2_‐KI also showed lower viability in response to heat stress (Feng et al. 2018). 2,3,5‐Triphenyltetrazolium chloride (TTC) solution is also a chromogenic dye used to stain pollen, which colors live pollen red. TTC‐stained cotton pollen showed lower viability under drought stress in two cultivars of varying drought tolerance (Hu et al. 2020). Alexander's stain is another common chromogenic method used to distinguish between viable and aborted pollen, which stains viable pollen dark blue, while aborted pollen stains are colored light blue (Alexander 1969).

**TABLE 1:** Overview of the most important staining, clearing, and imaging techniques used to evaluate stress responses in reproductive tissues.

Type of change to be analyzed	Method	Organ/tissue	References
Morphological change	TUNEL	Anther, tapetum	(Feng et al. 2018) (Zhao, Guan, et al. 2023) (Lyu et al. 2019)
	SCRI Renaissance	Ovule, whole seeds, embryos	(Musielak et al. 2015)
	Whole seed surface measurement	Whole dry seed	(Koh et al. 2022) (Jamil et al. 2017) (Ebrahimi Naghani et al. 2024)
	OCT	Seed interior, excluding endosperm	(Ravichandran et al. 2017)
	MRI	Seed coat, endosperm, embryo	(Borisjuk et al. 2012) (Rolletschek et al. 2021)
	(Whole‐mount) chloral hydrate‐based clearing	Ovule, seeds	(Kurihara et al. 2015)
Physiological change	ROS staining (e.g., CM‐H_2_DCFDA, DHE, PO1, CellROX)	Anther, pollen grains, pollen tubes, ovules, stigma	(Muhlemann et al. 2018) (Postiglione et al. 2024) (Ouonkap et al. 2024) (Santiago and Sharkey 2019) (Sun et al. 2005) (Sankaranarayanan et al. 2025) (Do et al. 2019)
	Ca^2+^ imaging (e.g., GCaMP6m, YC3.6)	Pollen grains, pollen tubes, ovules	(Pei et al. 2024) (Barberini et al. 2018) (Iwano et al. 2012)
Transcriptional and translational change	Whole‐mount in situ hybridization	Whole seed, embryo	(García‐Aguilar et al. 2005) (Páldi et al. 2020) (Yang et al. 2001) (Ghosh Dastidar et al. 2016)
	F‐WISH	Whole seed, embryo	(Bleckmann and Dresselhaus 2016)
	M2WISH	Whole seed, embryo	(Chelysheva et al. 2024)
	smRNA‐FISH	Anther, embryo	(Zhao, Fonseca, et al. 2023)
	Immunocytochemistry	Whole seed, seed coat, embryo	(Sauer et al. 2006) (Macquet et al. 2007) (Nagaki et al. 2017)
Metabolic change	NMR‐based CSI	Embryo, seed coat, endosperm	(Neuberger et al. 2008)
	Flavonols (DPBA)	Pollen grain, pollen tube	Postiglione et al., 2024 (Postiglione et al. 2024) (Muhlemann et al. 2018)
	Cell wall (e.g., calcofluor, aniline blue, propidium iodide, JIM antibodies)	All reproductive tissues	

Fluorescent dyes, such as the dual staining method with fluorescein diacetate (FDA) and propidium iodide (PI), are more commonly used (Figure 1C). FDA is a live pollen sensor that requires the presence of cellular esterase activity in live pollen to cleave the acetate groups of FDA to generate fluorescein, a green fluorescent product. Co‐staining with PI allows for the detection of dead pollen grains because the dye is membrane impermeable and is only able to enter dead cells through their ruptured membrane. The FDA/PI co‐staining provides a more accurate measurement of pollen viability than chromogenic dyes because it requires a metabolically active cell, and the co‐staining makes the distinction between live and dead less subject to bias by the experimenter. In fact, FDA staining alone showed a high correlation between pollen viability and in vitro pollen germination in date palm, especially compared to the chromogenic stains I_2_‐KI and Alexander's stain, which showed very low correlation by overestimating the number of viable pollen grains (Al‐Najm et al. 2021). FDA/PI dual‐stained pollen grains of tomato showed lower pollen viability when pollen development occurred at high temperature (Muhlemann et al. 2018). FDA/PI co‐staining of pollen grains of the tropical crop *Jatropha curcas* showed decreased viability after pollen grains were exposed to in vitro heat stress. Moreover, the decrease in viability was dependent on the temperature and the duration of the heat stress treatment (Kasthurirengan et al. 2025).

**FIGURE 2:**
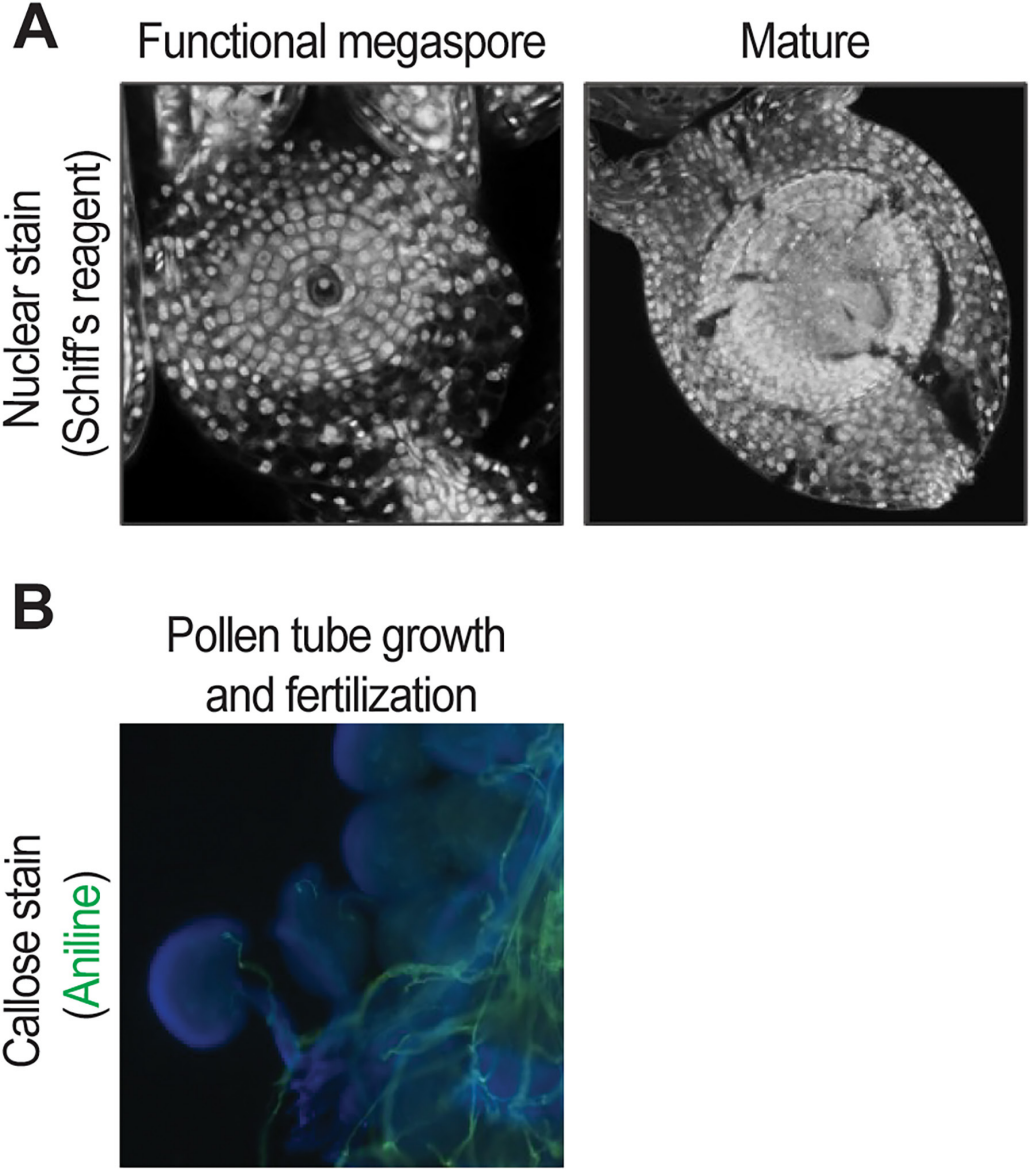
Imaging approaches to monitor ovule development and fertilization. (A) Embryo sacs of *Eragrostis curvula* in the functional megaspore and mature stages, stained with Schiff's reagent and imaged using CLSM (Pasten et al. 2025). (B) In vivo pollen tube and fertilization analysis in *A. thaliana* , using aniline blue staining (shown in green) and epifluorescence microscopy. Ovules are depicted in blue. Image by Mendes and Pasten (unpublished manuscript).

In addition to the viability of pollen grains, pollen germination and pollen tube growth are also greatly affected by abiotic stresses. In vivo pollen tube growth can be visualized and analyzed by staining with aniline blue, a fluorescent dye that stains callose (Figure 2B). For example, aniline blue‐stained pollen tubes inside the pistil experienced reduced elongation in heat‐sensitive tomato cultivars when subjected to high temperature (Ouonkap et al. 2024). In rice spikelets of a heat‐sensitive mutant exposed to high temperature, aniline blue‐stained pollen tubes were only found in the style, compared to the heat‐tolerant wild type, where the pollen tubes were detected in the style and ovary, indicating reduced tube growth under heat stress in the heat‐sensitive mutant (Zhang et al. 2018). Similarly, aniline blue‐stained styles of cotton showed decreased pollen tube growth under drought stress (Hu et al. 2019).

Unlike in vivo pollen tube growth measurement, in vitro measurement can be visualized by brightfield microscopy without staining (Figure 1D). Decreased pollen tube growth under heat stress is highly correlated to fruit/seed set under heat stress (Ouonkap et al. 2024), which makes assessment of pollen tube growth a crucial indicator of reproductive thermotolerance. Although in vitro pollen tube elongation decreases under high temperatures, cold stress applied to rice spikelets has been shown to increase pollen tube elongation without affecting germination rate in an in vitro study (Parrotta et al. 2019). Testing the effect of different temperatures on pollen germination and tube growth can be simultaneously performed using a thermal cycler with adjusted gradient temperature and incubation time as a high‐throughput technique (Zhao et al. 2025). Live imaging of pollen tube growth directly under heat stress conditions is also possible through a heated stage setup that is added to the microscope (Kahrizi et al. 2025). Additionally, quantification of in vitro pollen tube growth via live imaging can be done in a high‐throughput manner by partial automation of the analysis with software called TubeTracker (Ouonkap et al. 2025).

### 2.2 Ovule Development

Imaging methods have become fundamental tools for understanding how abiotic stress impacts ovule development at the structural and cellular levels. Traditional approaches such as histological sectioning, bright field microscopy, and callose staining with aniline blue remain widely used to detect ovule abortion, intracellular disorganization, plastid degradation, and embryo sac collapse, all of which are characteristic markers of stress‐induced developmental failure (Chiluwal et al. 2020; Li et al. 2023; Sun et al. 2004; Wang et al. 2021). In particular, callose (β‐1,3‐glucan) deposition serves as an important morphological indicator since its abnormal accumulation is observed in aborted ovules and correlates with impaired gametophyte maturation.

Confocal laser scanning microscopy (CLSM) has been one of the most powerful tools to characterize ovule architecture under both normal and stress conditions. By combining CLSM with specific staining reagents such as Schiff's reagent (Figure 2A), SCRI Renaissance, TO‐PRO‐3, or propidium iodide, researchers have been able to generate detailed three‐dimensional reconstructions of ovules, allowing the assessment of structural alterations across developmental stages (Bink et al. 2001; Cornaro et al. 2025; Mendocilla‐Sato et al. 2017; Pasten et al. 2025; Reyes‐Olalde et al. 2015). Clearing techniques and whole‐tissue preparations (Attuluri et al. 2022; Kurihara et al. 2021) further improve visualization by providing intact images of ovule morphology even when tissue integrity is compromised by environmental stress.

High‐resolution electron microscopy has also been central to stress studies. Scanning electron microscopy (SEM) has revealed that heat stress modifies ovule anatomy, reduces tissue compactness, and correlates with decreased seed set, as observed in sorghum (Djanaguiraman et al. 2018). Synchrotron‐based phase contrast x‐ray imaging, in combination with fluorescence microscopy, has enabled the visualization of pollen tube growth inside ovules, showing that heat stress not only reduces pollen viability but also compromises ovule receptivity in pea (Jiang et al. 2019). These approaches provide unique opportunities to connect environmental stress with structural consequences in female reproductive tissues.

Overall, the integration of classical and advanced imaging methods highlights the spectrum of morphological responses of ovules to abiotic stress, ranging from callose accumulation and cell disorganization to large‐scale alterations in tissue differentiation and fertilization efficiency.

### 2.3 Seed Development

The seed develops following the fertilization of the egg cell and central cell by the two sperm cells, triggering the formation of the embryo and endosperm, protected by a seed coat. The endosperm stores the nutrients for the embryo and is completely absorbed by the embryo at maturity. In grains and cereals, however, the endosperm persists during embryo development, becomes the major storage site for starch and protein (Taiz et al. 2023), and will be the edible part of the grain. The endosperm also controls seed size (Li and Berger 2012). The seed coat acts as a protective layer for the seed and regulates seed dormancy. The innermost layer of the integuments develops into the endothelium, which produces proanthocyanidin, the precursor of tannins (Debeaujon et al. 2017). The embryo develops in two phases, morphogenesis and maturation (Verma et al. 2022). During the morphogenesis stage, the embryo acquires all morphogenetic features of the future germinating seedling, that is, cotyledons, shoot apex, hypocotyl, root and root apex, and grows to occupy the space in the seed cavity. During the maturation stage, the embryo stops its growth and transitions to maturation, which, in dicotyledonous species, means the accumulation of storage lipids and proteins. Abiotic stress during seed development may impact the various tissues (seed coat, endosperm, and embryo) differently at different developmental stages (morphogenesis, maturation, desiccation) and have consequences for seed viability and nutritive properties. Thereby, imaging the impact of stress in seeds or seed tissues may provide valuable structural and physiological information for post‐fertilization fitness.

**FIGURE 3:**
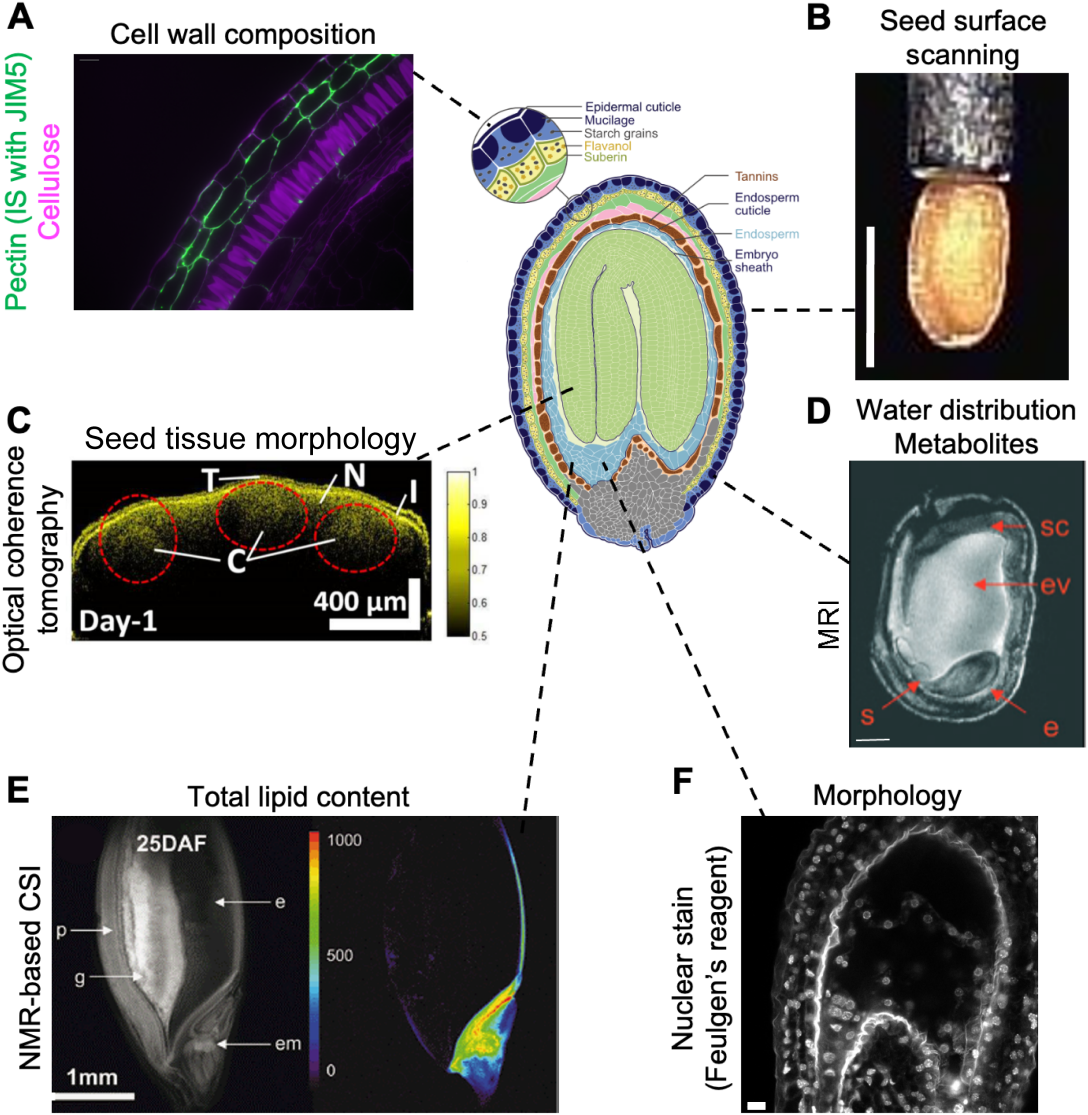
Imaging methodologies in seeds. Schematic image of *Arabidopsis thaliana* seed. Modified from Verma et al. (2022). (A) Immunostaining of *Brassica napus* seed at 18 days after pollination. Magenta indicates cellulose stained with Calcofluor White, green indicates pectin immunodetected with JIM5 and anti‐rat IgG Alexa 488. Scale bar = 20 μm. Courtesy of Unnikannan Prabhullachandran (unpublished manuscript). (B) Dry seed phenotype of *Arabidopsis thaliana*, Col‐0. Scalebar indicates 0.5 mm. Modified from Ebrahimi Naghani et al. (2024). (C) 2D swept source optical coherence tomography image of a *Capsicum annuum* seed showing cotyledons (C), the inner seed coat (I), the non‐micropylar endosperm (N) and the testa (T). The dashed red circles show the enclosed cotyledon regions. Modified from Ravichandran et al. (2017). (D) Longitudinal section from MRI of *Pisum sativum* seed. Indicated are the embryo (e), the endospermal vacuole (ev) and the suspensor (s). Scale bar = 1 mm. Reprinted from Borisjuk et al. (2012). (E) Quantitative imaging of lipids in a living barley ( *Hordeum vulgare* ) grain using NMR‐based CSI. The grain structure at mid‐storage stage is presented on the left. The total lipid content (μmol/g fresh weight), shown on the right, is color‐coded. DAF, days after flowering; e, endosperm; em, embryo; g, grease region; p, pericarp. Reprinted from Neuberger et al. (2008). (F) Feulgen‐stained *Arabidopsis thaliana* seed. Scale bar = 10 μm. Courtesy of Oussama Guennich (unpublished).

The size of dried seeds can be determined by measuring the seed surface area, maximum seed length, and width using a device called Boxeed from Labdeer s.r.o. (Koh et al. 2022). This tool is particularly useful for the high‐throughput and high‐resolution seed size measurement of dry seeds (Figure 3B). It is also possible to determine the seed shape and color. However, since it only scans the surface of the seeds, the determination of the internal seed morphology and the monitoring of embryo development are not possible. Seed surface changes have so far only been reported for heat stress in *Arabidopsis* (Ebrahimi Naghani et al. 2024) and wheat (Jamil et al. 2017).

To non‐invasively monitor morphological changes inside seeds, techniques such as optical coherence tomography (OCT) (Ravichandran et al. 2017) or magnetic resonance imaging (MRI) (Borisjuk et al. 2012; Rolletschek et al. 2021) can be used (Figure 3C,D). OCT is based on changes in the refractive index of scattering layers within the sample. Using OCT, Ravichandran et al. (2017) monitored seed growth and the state of germination under salt stress in *Capsicum annuum* and observed a slower growth rate of the seeds in stressful conditions. However, OCT could not resolve the endosperm, which can be overcome with NMR (nuclear magnetic resonance) or MRI. MRI is based on the theory of NMR to acquire images. The term “NMR” is sometimes used to refer both to in vitro experiments and to MRI outside of the medical field (Boesch 1999; Borisjuk et al. 2012; Khashami 2024). For both methods, three orthogonal magnetic fields are generated and atoms having no spin (non‐zero nuclear magnetic moment) are detected, usually ^1^H, ^13^C, ^19^F, ^23^Na, ^31^P, and ^39^K (Borisjuk et al. 2012). Melkus et al. (2009) reconstructed a digital model with MRI, which allowed them to visualize seed anatomy in three dimensions (3D), measure seed organ volume and size, and assess the embryo‐endosperm ratio. MRI has also been applied to detect changes in various plant tissues and plant species during cold and drought stress (Borisjuk et al. 2012). However, this method is not suitable for long‐term monitoring because it requires radioactive labeling, and it has only a limited spatial resolution. Alternative methodologies for obtaining structural information in the seed are Feulgen staining (Figure 3f; Braselton et al. 1996) and Hoyer's medium clearing based on chloral hydrate, in which samples are fixed and imaged by CLSM or with a bright field microscope equipped with differential interference contrast (DIC) optics, respectively.

## 3 Reactive Oxygen Species in Reproduction Under Abiotic Stress

### 3.1 Approaches to Imaging Reactive Oxygen Species, Small Molecules With Important Functions in Development and Stress Responses

A hallmark of abiotic stress responses is the increased production of ROS. These oxygen‐derived molecules exhibit highly reactive properties that can cause oxidative damage to cellular macromolecules when present at high concentrations. Consequently, this has negative effects on diverse physiological and developmental processes. While ROS were traditionally regarded exclusively as harmful by‐products of different metabolic processes, they are now known to also act as vital secondary messengers driving important developmental and physiological processes such as root development, stomatal closure, flower and meristem development, reproduction, seed after‐ripening and germination, and hormone signaling (Chapman et al. 2019; Martin et al. 2022; Mhamdi and Van Breusegem 2018). ROS act as secondary messengers in signaling by reversibly oxidizing cysteine residues of proteins, which modulate the structure and function of these proteins. To tightly regulate ROS levels and prevent oxidative damage, plants have diverse enzymatic and non‐enzymatic antioxidant machineries, such as peroxidases, flavonoids, and ascorbate, which balance ROS levels and prevent oxidative stress (Chapman et al. 2019; Gechev and Petrov 2020). ROS are synthesized by a small network of enzymes localized in different cellular compartments (reviewed by Chapman et al. 2019; Martin et al. 2022). They include superoxide O_2_^−^, hydrogen peroxide H_2_O_2_, hydroxyl radical (OH^−^), singlet oxygen ^1^O_2_, which have varied levels of both stability and reactivity.

Given the important roles of ROS as both signaling molecules and stress response molecules, different techniques have been developed and utilized to monitor their presence both spatially and temporally in plant tissues. Histochemical, colorimetric staining methods have been utilized in the past and continue to be used for ROS detection. These include stains such as nitro blue tetrazolium chloride (NBT), which is a dye that reacts with superoxide and is reduced in the cell to produce the purple‐colored product formazan (Grellet Bournonville and Díaz‐Ricci 2011). Additionally, 3,3′‐diaminobenzidine (DAB) is a dye that reacts with hydrogen peroxide to produce a brown colored chromogen that can also be viewed under bright field microscopy (Jambunathan 2010). Although these stains provide qualitative or semi‐quantitative insights, they are limited in sensitivity and spatial resolution.

Since histochemical staining can generally only be used as a qualitative assessment of ROS levels and localization, fluorescent dyes have emerged as an alternative method that can provide a quantitative measurement of ROS at a higher (subcellular) resolution (Ortega‐Amaro et al. 2016). A commonly used fluorescent dye is 2′,7′‐dichlorodihydrofluorescein diacetate (H_2_DCFDA), which permeates the cell membrane in a quenched state and, upon entry, is de‐esterified to H_2_DCF by esterases, which reacts with ROS and is then oxidized to form the fluorescent DCF molecule (Eruslanov and Kusmartsev 2010). CM‐H_2_DCFDA is a chloromethyl derivative that allows better retention of the dye inside the cell for longer‐term imaging. Because oxidation is achieved by a reaction with more than one type of ROS, this dye is considered a general ROS sensor. Like CM‐H_2_DCFDA, CellROX is another general ROS sensor that enters the cell in a non‐fluorescent or weakly fluorescent state and, upon oxidation by ROS, produces a stable fluorophore (Lozoya‐Gloria et al. 2017). Some species of ROS can be measured with ROS‐specific dyes. Superoxide can be measured by dihydroethidium (DHE), which reacts with intracellular O_2_^−^ to produce the fluorescent product 2‐hydroxyethidium (2HE) (Zhao et al. 2003). The boronate‐based Peroxy Orange 1 (PO1) is a highly selective and sensitive H_2_O_2_ sensor, which, upon entering the cell, reacts with H_2_O_2_ to produce a fluorescent signal (Dickinson et al. 2010). Amplex Red is also widely used to measure H_2_O_2_, especially in extracellular spaces where peroxidase catalyzes the one‐proton oxidation of Amplex Red via H_2_O_2_ to produce the fluorophore resorufin (Dębski et al. 2016). While these fluorescent dyes can relatively easily permeate cell membranes and have the advantage of measuring specific ROS, some have stability issues and are not appropriate for time course ROS measurements in response to stress or other treatments due to their irreversible oxidation (Passos and Saraiva 2019).

Genetically encoded fluorescent protein sensors have been developed to circumvent these limitations. For example, the HyPer molecular probe can detect H_2_O_2_ levels in real time through its inherent design, which is composed of the *E. coli* transcriptional regulator OxyR, specifically its regulatory domain Oxy‐RD that selectively senses H_2_O_2_ (Lyublinskaya and Antunes 2019). The sensing is based on the reaction between H_2_O_2_ and cysteine residues in the regulatory domain Oxy‐RD, which leads to a conformational change. Integration of a yellow fluorescent protein (YFP) into the region of conformational change leads to a change in the excitation wavelength following reaction with H_2_O_2_. The reversible nature of the change in excitation wavelength allows HyPer to sense dynamic changes in H_2_O_2_ levels in real time. Another variant of a genetically encoded ROS biosensor is the redox‐sensitive GFP (roGFP), which was developed by changing specific amino acid residues in regions close to the chromophore, allowing a redox‐sensitive GFP to be generated. The sensitivity is based on the detection of changes in the cellular redox state through a ratiometric shift in excitation peaks (Hanson et al. 2004).

### 3.2 ROS Dynamics During Reproduction Under Stress: Evidence From Imaging‐Based Approaches

#### 3.2.1 Pollen Development

Pollen development is particularly susceptible to high temperature and drought stress, which are known to trigger excess ROS accumulation (Gechev and Petrov 2020; Santiago and Sharkey 2019). For instance, imaging of ROS with DCFH‐DA (a dye similar to H_2_DCFDA) in rice whole anthers showed higher fluorescence levels in response to heat stress (Zhao et al. 2018; Zhao, Guan, et al. 2023). Whole rice anthers stained with DCFH‐DA also show higher fluorescence intensity under heat stress (Feng et al. 2018). In common bean, H_2_O_2_ extracted from whole anthers was measured by Amplex Red, which showed higher H_2_O_2_ levels in a heat‐tolerant cultivar compared to a heat‐sensitive cultivar (Santiago et al. 2021). Moreover, the same study showed increased Amplex Red fluorescence signal in the anther extract of the heat‐sensitive cultivar in response to high temperature stress. CM‐H_2_DCFDA staining of tomato ( *Solanum lycopersicum* ) of both pollen grains and pollen tubes also showed elevated ROS levels under short‐term high temperature stress (Muhlemann et al. 2018; Postiglione et al. 2024). Pollen tubes of heat‐tolerant tomato cultivars stained with CM‐H_2_DCFDA showed lower ROS levels under optimal and high temperatures compared to less heat‐tolerant cultivars (Ouonkap et al. 2024). All cultivars tested in the study showed an increased fluorescence under heat stress, especially at the tip of the growing tube, suggesting localized ROS accumulation (Ouonkap et al. 2024). Real‐time measurement of H_2_O_2_ in pollen grains and anthers has yet to be studied; however, the roGFP‐ORP1 and the HyPer sensor are promising tools for measuring the effects of different abiotic stresses (Ugalde et al. 2021; Nietzel et al. 2019). For instance, the model plant *Arabidopsis thaliana* transformed with the HyPer molecular probe was used to detect changes in H_2_O_2_ levels in root tips exposed to aluminum (which inhibits root growth when present in the soil). The HyPer sensor showed a dose‐dependent decrease in H_2_O_2_ levels in roots exposed to different concentrations of aluminum (Hernández‐Barrera et al. 2015). The HyPer probe can be expressed by the pollen‐specific promoter *LAT52* (Lara‐Rojas et al. 2023) for potential dynamic H_2_O_2_ measurements in pollen grains under stress conditions. roGFP can also be expressed under the *LAT52* promoter (Huang and Tang 2015) to measure dynamic ROS changes. In fact, the newly developed roGFP2‐PRXIIB is pH‐insensitive and shows a fast oxidation rate compared to its predecessor (Hu et al. 2025). Pollen tubes of tomato transformed with roGFP‐PRXIIB under the *LAT52* promoter were treated with rapid alkalinization factors (RALFs) (which trigger ROS burst) and showed higher roGFP2‐PRXIIB signal compared to mock treatment (Hu et al. 2025), thus validating roGFP as a valuable tool for real‐time ROS measurement in stressed pollen.

#### 3.2.2 Ovule and Seed Development

To our knowledge, there are few studies imaging ROS dynamics under abiotic stress during ovule development and fertilization. However, there is evidence that ROS play a role in these developmental processes. H_2_DCFDA staining of *A. thaliana* ovules showed increased fluorescence intensity after salt stress; the intensity was dependent on the duration of the stress, and the fluorescence was also localized in different regions of the ovule (Sun et al. 2005). In the seed, ROS accumulation was monitored during seed development using histochemical staining as described above for pollen, for example, in the whole seed and endosperm of *Zea mays* (Zang et al. 2024), as well as in the seed coat of *Avena fatua* (Cembrowska‐Lech 2020). ROS generation as a response to silver nanoparticle treatment was measured with DCFH‐DA staining in whole maize seeds using a fluorescent whole plant imaging system (Chen et al. 2023). This showed increased ROS in the presence of silver nanoparticles, which was suggested as a mechanism for stress priming that allowed for better seed germination under stress conditions. The monitoring of ROS in the embryo has not been done so far, probably due to the absence of a biosensor expressed in seeds. A solution may be to express the HyPer probe under ovule‐, seed‐, embryo‐, endosperm‐, and seed coat‐specific promoters (Table 2).

**TABLE 2:** Overview of the most important tissue‐ or cell‐specific promoters for use in reproductive tissues.

Organ/tissue	Promoter	References
Tapetum	pA9	(Paul et al. 1992)
Pollen tube	pLAT52	(Twell et al. 1990)
Ovule (nucellus, chalaza, integuments)	pSTK	(Matias‐Hernandez et al. 2010) (Pinyopich et al. 2003)
Ovule (MMC)	pKNU	(Tucker et al. 2012)
Ovule (FM)	pLC2	(Di Fino et al. 2017) (Pessino et al. 2024)
Ovule (MMC, FM, integuments)	pARF10	(Liu et al. 2007) (Pessino et al. 2024)
Ovule, shoot apical meristem	pWUS	(Plong et al. 2021) (Mayer et al. 1998)
Ovule (synergid cells)	pMYB98	(Kasahara et al. 2005) (Drews and Koltunow 2011)
Ovule, pre‐globular embryo	pWOX2	(Liao and Weijers 2018) (Breuninger et al. 2008) (De Storme et al. 2016)
Post‐globular embryo	pRPS5A	(Liao and Weijers 2018) (Weijers et al. 2001)
Testa	pBANUYLS	(Debeaujon et al. 2003)
Endosperm	pFWA	(Kinoshita et al. 2004)

## 4 Exploring Calcium Signaling in Plants: Techniques and Roles in Reproduction Under Stress

### 4.1 Imaging Tools for Calcium Dynamics in Plants

Calcium (Ca^2+^) is a ubiquitous second messenger that regulates diverse physiological and developmental processes in plants. In plant cells, free Ca^2+^ is compartmentalized within intracellular stores and in the apoplast, while cytosolic Ca^2+^ concentrations are maintained at low levels (around 100 nM) to prevent cytotoxicity. Ca^2+^ homeostasis is finely regulated by active transporters, which sequester Ca^2+^ into storage compartments, and by Ca^2+^‐permeable channels, which mediate passive influx into the cytosol (Costa et al. 2023; Grenzi et al. 2021). The precise spatial and temporal regulation of these proteins shapes dynamic Ca^2+^ changes, which are subsequently decoded to activate specific downstream processes.

Imaging technologies have dramatically transformed our understanding of Ca^2+^ dynamics by enabling real‐time visualization of fast, transient, and spatially resolved signals in living cells (Grenzi et al. 2021; Sheng et al. 2024). Historically, Ca^2+^ measurements relied on indirect or destructive biochemical assays, which lacked spatial or temporal resolution. However, two major classes of Ca^2+^ indicators, chemically synthesized dyes and genetically encoded indicators, now allow for direct and dynamic monitoring of Ca^2+^ fluxes in plant cells (Ghosh et al. 2023; Grenzi et al. 2021; Oak and Mali 2024).

Chemical Ca^2+^ indicators are small fluorescent molecules based on the BAPTA scaffold (a high‐affinity Ca^2+^ chelator derived from EGTA), which confers high selectivity for Ca^2+^ over Mg^2+^. Widely used indicators include Fura‐2, Indo‐1, Fluo‐3, Fluo‐4, and Calcium Green‐1 (Ghosh et al. 2023; Oak and Mali 2024; Sheng et al. 2024). These dyes are typically loaded into cells as acetoxymethyl esters (AM esters), which passively diffuse across membranes and are subsequently activated by cytosolic esterases (Booth et al. 2016). Alternatively, direct microinjection of the free acid form enables targeting specific subcellular compartments. Upon binding calcium, these dyes either increase in fluorescence intensity or exhibit spectral shifts, enabling both qualitative and quantitative imaging of intracellular Ca^2+^.

Though originally developed for animal cells, where they revealed subcellular phenomena like Ca^2+^ sparks and waves, chemical indicators have since been successfully adapted for plant systems. In particular, they have been employed to study cytosolic and organellar Ca^2+^ responses to mechanical, osmotic, and salt stress (Sheng et al. 2024). While they lack the cell‐type specificity and genetic versatility of protein‐based tools, their high sensitivity and ease of application continue to make them valuable for studies in non‐genetically tractable systems.

The development of Genetically Encoded Ca^2+^ Indicators (GECIs) has addressed many of the limitations of chemical dyes. Tools such as the Yellow Cameleon (e.g., YC3.6) enable ratiometric FRET‐based detection of Ca^2+^ changes in living tissues with cell‐type and organelle specificity (Bonza et al. 2013; Ghosh et al. 2023; Iwano et al. 2009; Krebs et al. 2012; Loro et al. 2012, 2016). Expressed under tissue‐specific promoters, these indicators provide powerful insight into developmental processes like pollen tube guidance and fertilization. Moreover, modern imaging platforms, including confocal laser scanning, light sheet, and spinning disk microscopy, have significantly improved the spatiotemporal resolution of GECI‐based imaging while minimizing photobleaching (Ghosh et al. 2023; Himschoot et al. 2018).

To improve interpretability and precision, advanced Ca^2+^ imaging setups often integrate dual‐channel systems, kymograph analysis, and pharmacological treatments to dissect the roles of specific channels, pumps, or intracellular stores (Barberini et al. 2018; Shang et al. 2005). These integrated approaches extend the scope of calcium imaging from single‐cell analysis to organ‐level contexts, supporting applications in both plant and animal biology.

The broader field has also benefited from the use of bioluminescent sensors like aequorin, originally derived from jellyfish (Shimomura 2005). Aequorin has been widely employed in transgenic plants to study Ca^2+^ responses to stress signals such as pathogen elicitors and abiotic stresses (Ghosh et al. 2023; Zhang et al. 2015). Though limited by weak luminescence, aequorin's utility has been expanded through hybrid indicators like G5A, which combines aequorin with GFP to improve brightness and spatial resolution (Xiong et al. 2014). Meanwhile, advances in microscopy, such as Single‐Plane Illumination Microscopy (SPIM) and deconvolution microscopy, have facilitated three‐dimensional imaging of complex tissues, enhancing our ability to resolve intracellular calcium dynamics (Candeo et al. 2017; Costa et al. 2013; Pnevmatikakis et al. 2016).

While chemical dyes such as Fura‐2 and Indo‐1 provide rapid response kinetics and are highly sensitive to Ca^2+^ variations, they are often invasive and not ideal for long‐term studies. GECIs, by contrast, offer the advantage of sustained, non‐invasive imaging in intact systems, though with slower kinetics. The decision between these tools thus depends on experimental needs, short‐ or long‐term imaging, subcellular targeting, or genetic accessibility of the model organism (Grenzi et al. 2021; Sheng et al. 2024).

In summary, advances in calcium imaging, from the early use of synthetic dyes to the development of sophisticated GECIs and imaging platforms, have vastly expanded our capacity to observe and quantify Ca^2+^ signals in living systems. These tools continue to reveal calcium's role as a central regulator of cellular function, development, and environmental response in both plants and animals.

### 4.2 Calcium Signaling in Plant Reproduction Under Stress: From Pollen Hydration to Fertilization

**FIGURE 4:**
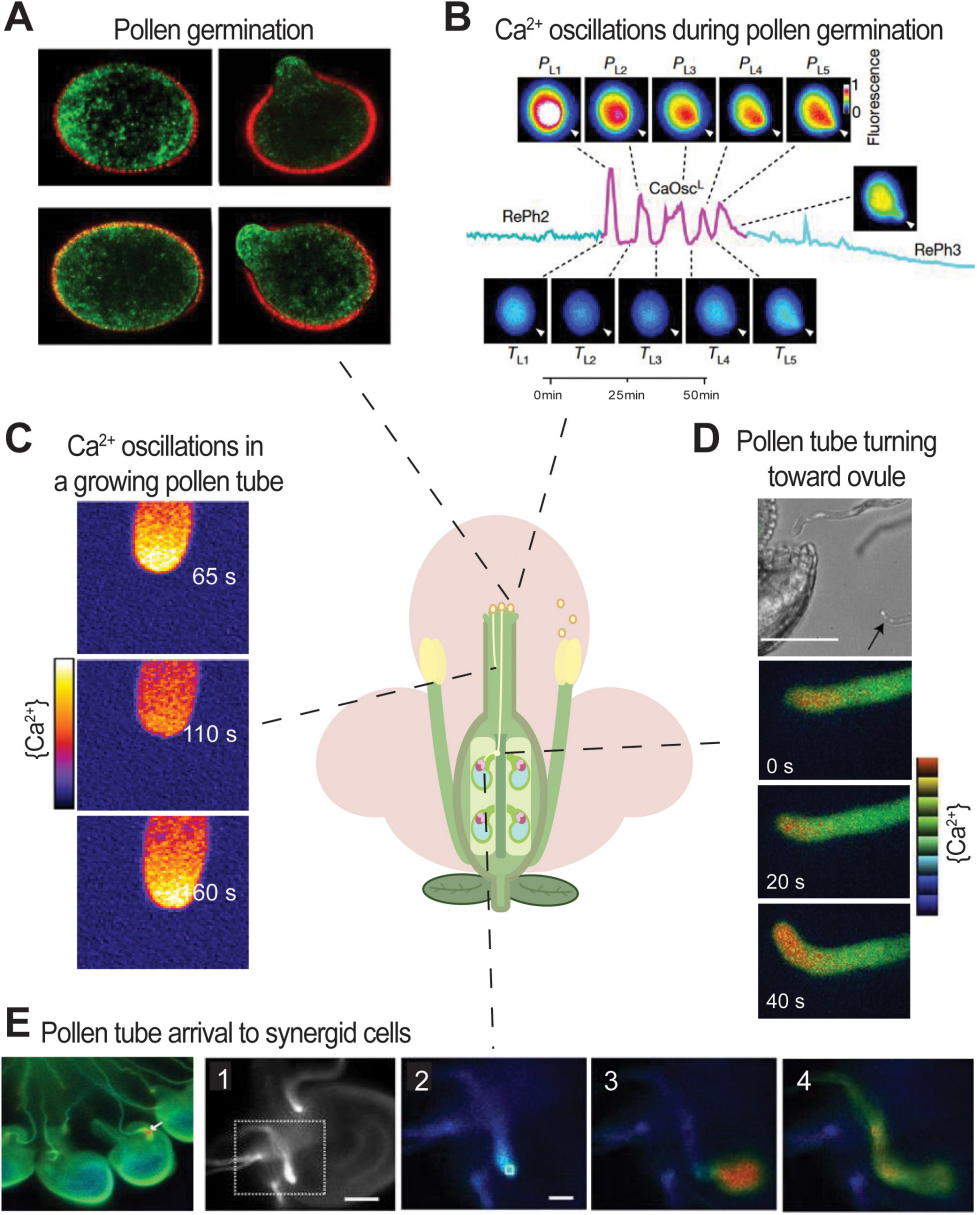
Ca^2+^ dynamics and localization during pollen germination and tube growth. (A) Plasma membrane localization of OSCA2.1 and OSCA2.2 in pollen. Fluorescence of OSCA2.1–GFP, OSCA2.2–GFP, and the membrane marker FM4‐64 in pollen grains placed on germination medium for 0 (left panels) or 120 min (right panels), imaged using CLSM. Adapted from Pei et al. (2024). (B) GCaMP6m fluorescence traces showing intracellular Ca^2+^ oscillations in wild‐type pollen grains (expressing pLAT52::GCaMP6m) prior to germination. Resting phases (RePh2–3) are interrupted by large oscillations (CaOsc^L^). Arrowheads indicate the germination aperture. PL, peak CaOsc^L^; TL, trough CaOsc^L^. Adapted from Pei et al. (2024). (C) Pollen tube growth and Ca^2+^ dynamics in tomato plants expressing YC3.6 under the pollen‐specific promoter LAT52. Time‐lapse imaging of in vitro growing pollen tube. Fluorescence ratio images (pseudo‐color) reveal a tip‐focused [Ca^2+^]_cyt_ gradient. Adapted from Barberini et al. (2018). (D) Ratiometric images of pAct1::YC3.60‐expressing pollen tubes growing toward functional ovules. The pollen tube tip (arrow) turns toward a wild‐type ovule. Scale bar: 5 μm. Adapted from Iwano et al. (2012). (E) Increase in [Ca^2+^]_cyt_ in synergid cells after pollen tube arrival. Ratiometric images of an Arabidopsis pAct1::YC3.6‐expressing line prior (1) and during pollen tube arrival at the synergic cells (2–4). The white arrow indicates the [Ca^2+^]_cyt_ increase in synergid cells. Adapted from Iwano et al. (2012).

During plant reproduction, Ca^2+^ plays a pivotal role in regulating processes from pollen hydration to sperm delivery. Upon pollen grain hydration and germination, extracellular Ca^2+^ influx is mediated by various plasma membrane‐localized channels, including stretch‐activated (SA) channels and hyperpolarization‐activated Ca^2+^ channels (HACCs) (Figure 4A,B; Pei et al. 2024). Using *A. thaliana* pollen grains expressing the GCaMP6m Ca^2+^ indicator in WT, *osca2.1*, *osca2.2* single mutants, and the *osca2.1/2.2* double mutant, the involvement of mechanosensitive OSCA channels in Ca^2+^ oscillations preceding pollen germination has been demonstrated. This finding underscores the role of Ca^2+^ signaling in pollen tube initiation (Figure 4A,B; Iwano et al. 2004; Pei et al. 2024).

Following successful germination, pollen tubes exhibit a tip‐focused Ca^2+^ gradient that is essential for polarized growth. Earlier studies using aequorin and more recent works employing GECIs have demonstrated a strong correlation between pollen tube elongation and cytosolic Ca^2+^ oscillations in *A. thaliana* . The characteristic pulsatile growth pattern of pollen tubes is regulated by a complex interplay of cytosolic Ca^2+^ influx, efflux, and intracellular mobilization (Iwano et al. 2004; Messerli et al. 2000). Comparable Ca^2+^ dynamics have also been observed in other plant species, such as *Lilium longiflorum* and *Solanum lycopersicum* . In these studies, Fura‐2‐dextran dye and the FRET‐based GECI YC3.6 were used to monitor cytosolic Ca^2+^ concentrations ([Ca^2+^]_cyt_) at the pollen tube tip and to visualize calcium gradients along the length of the growing tube (Figure 4C; Barberini et al. 2018; Pierson et al. 1996). Notably, Damineli et al. (2017) introduced an open‐source computational method to characterize oscillatory signatures in *A. thaliana* . This tool enabled high‐resolution analysis and synchronization assessment between Ca^2+^ oscillations, pollen tube growth, and proton fluxes with subpixel precision (Damineli et al. 2017).

Further insights into Ca^2+^‐dependent mechanisms have been gained through the use of advanced Ca^2+^ sensors. A particularly illustrative example comes from studies on cyclic nucleotide‐gated channels (CNGCs) and their role in pollen tube directional growth (Frietsch et al. 2007). CNGCs are activated by both cNMPs binding and intracellular Ca^2+^ binding, which regulate cyclic Ca^2+^ fluctuations (Pan et al. 2019). Importantly, the CNGC18 channel has been reported to play a prominent role in pollen tube growth since the *cngc18* mutation leads to male sterility (Gu et al. 2017). Indeed, *cngc18* mutant pollen tubes show severe defects in growing over long distances, with premature termination with burst events, and display non‐directional growth. Arabidopsis lines expressing the YC3.6 Ca^2+^ sensor in pollen tubes showed abnormal Ca^2+^ oscillation patterns, with irregular drifts in basal Ca^2+^ levels. Since regular oscillations in [Ca^2+^]_cyt_ are required for correct pollen tube growth, these irregular gradients might be responsible for pollen tube growth defects in the *cngc18* mutant (Gu et al. 2017). Importantly, the asymmetric localization of the CNGC18 protein, at the pollen tube‐tip lateral regions, supports the role of the protein in pollen tube guidance into the transmitting tract (Frietsch et al. 2007; Gao et al. 2016).

As the pollen tube approaches the embryo sac, live Ca^2+^ imaging of both pollen tubes and synergid cells expressing GECIs has revealed dynamic regulation of pollen guidance, including coordinated Ca^2+^ oscillations and a pronounced Ca^2+^ spike at the pollen tube tip immediately preceding tube rupture and sperm release (Figure 4D,E; Baillie et al. 2024; Desnoyer and Grossniklaus 2023; Ponvert and Johnson 2024). This Ca^2+^‐mediated crosstalk is regulated by the receptor‐like kinase FERONIA (FER), its co‐receptor LORELEI (LRE), and the Ca^2+^‐binding protein NORTIA (NTA). Disruption of any of these components, through genetic mutations, leads to altered Ca^2+^ signaling dynamics and ultimately causes fertilization failure (Baillie et al. 2024; Ponvert and Johnson 2024).

These conclusions were drawn from live‐imaging experiments that monitored the interaction between pollen tubes and female gametophyte cells: pollen tubes expressing *LAT52::dsRED* were visualized approaching synergid cells expressing the YC3.6 sensor (pMYB98:YC3.60) (Figure 4). Confocal microscopy enabled the detection of real‐time Ca^2+^ fluctuations within synergid cells during pollen tube arrival and rupture, revealing the essential role of FER, LRE, and NTA in coordinating this Ca^2+^‐dependent communication (Ponvert and Johnson 2024). Similarly, ACA9, a Ca^2+^‐ATPase localized to the pollen tube plasma membrane, is essential for pollen tube rupture. Loss of ACA9 function allows pollen tubes to reach the ovule but prevents sperm release, ultimately leading to male sterility (Figure 4; Iwano et al. 2012; Schiøtt et al. 2004).

Collectively, these findings highlight the central role of dynamic, spatially localized Ca^2+^ signals in orchestrating the fertilization process, from directional growth and guidance to intercellular communication and sperm release. The use of GECIs has been instrumental in revealing these mechanisms, and ongoing advances in imaging technologies promise to further unravel the complexity of Ca^2+^ signaling in plant reproduction.

Regarding Ca^2+^ signaling during seed and embryo development, the research is relatively scarce. Ca^2+^ accumulation has been determined in fixed wheat grains using LASER Ablation Inductively Coupled Plasma Mass Spectrometry (LA‐ICP‐MS) imaging (Wu et al. 2013), in which material ablated by laser is ionized and detected in the ICP‐MS (Becker et al. 2014). Wu et al. (2013) found enrichment of Ca^2+^ in the seed coat and aleurone, indicating the role of Ca^2+^ in structural maintenance. There is also evidence from *Oryza sativa* that the Ca^2+^‐dependent Protein Kinase 19 (OsCPK19) accumulates in developing seeds, while OsCPK23 is expressed in the seed endosperm and seems to control the biosynthesis of starch (DeFalco et al. 2010). These findings indicate that Ca^2+^‐responsive kinases contribute to key metabolic and developmental processes during seed formation. In addition, the NOD‐like receptor (NLR) ZAR1 has recently been implicated in early embryogenesis. It has been published that ZAR1 integrates extrinsic cues such as Ca^2+^ by interacting with calmodulin (CaM), and this Ca^2+^‐dependent regulation controls zygote division and the specification of apical and basal daughter cell fates (Yu et al. 2016). Overall, Ca^2+^ dynamics emerge as essential regulators of plant reproduction, yet many developmental roles remain to be uncovered.

## 5 Visualizing Transcriptional, Translational, and Metabolic Changes in Plant Reproduction Under Abiotic Stresses

Abiotic stresses result in the remodeling of the transcriptome, proteome, and metabolome. While imaging techniques have been developed to visualize and quantify transcripts, proteins, and certain metabolites, they are still rarely applied to reproductive tissues, especially in the context of stress. Below are various established techniques that could be more widely implemented to image stress responses during reproduction.

### 5.1 Transcriptional and Translational Responses to Abiotic Stress

**FIGURE 5:**
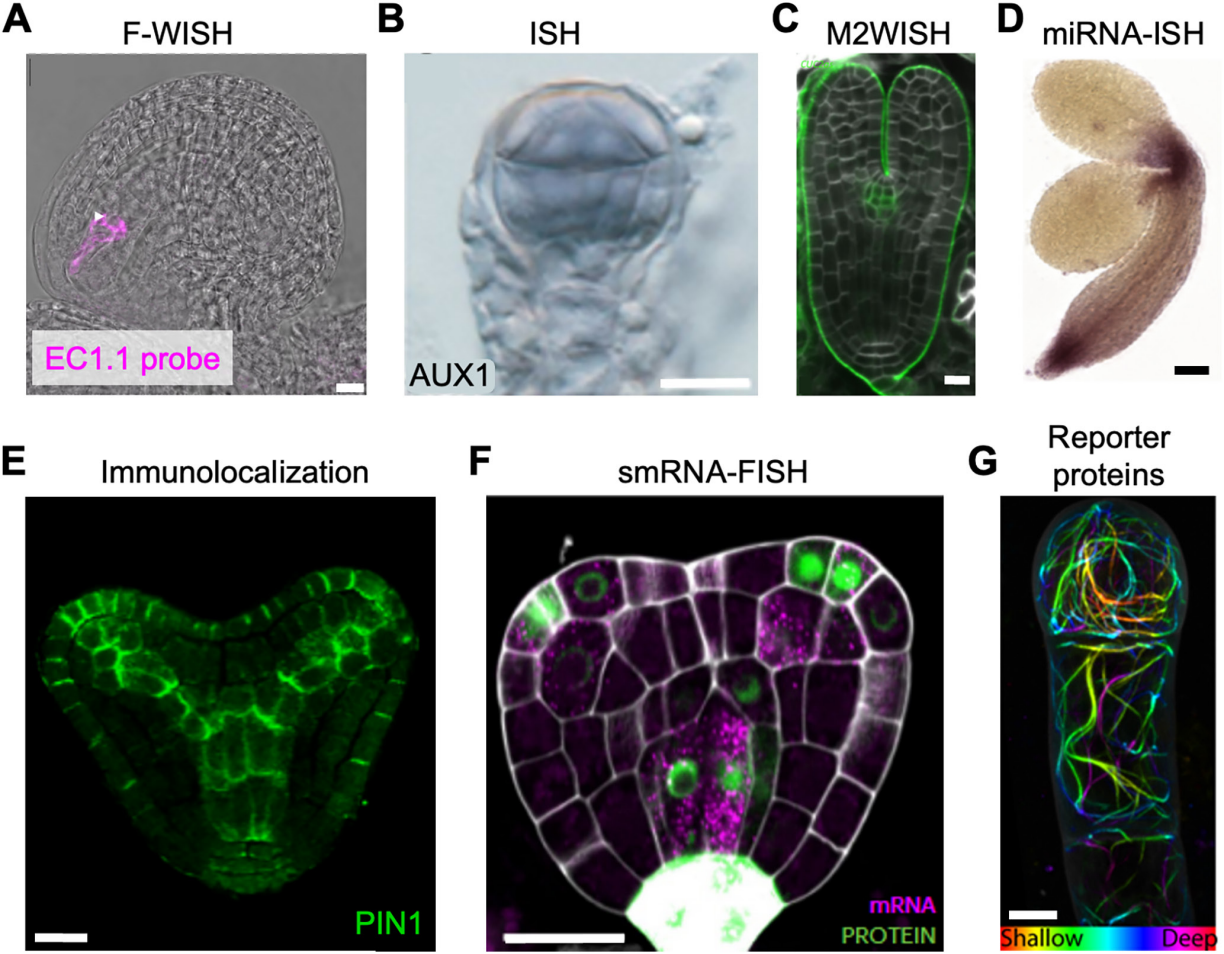
Imaging methods to visualize embryo development. (A) Visualization of EC1.1 mRNA (magenta) by F‐WISH. The arrowhead indicates the egg cell nucleus. Scale bar = 10 μm. Reprinted from Bleckmann and Dresselhaus (2016). (B) Expression of AUX1 (purple)visualized by RNA in situ hybridization. Scale bar = 20 μm. Adapted with permission from Robert et al. (2015). (C) Expression of *CUC2 mRNA* (green) in *tt4* mutant at the torpedo stage using M2WISH. Reprinted from Chelysheva et al. (2024). (D) Detection of miR390 miRNA (purple) using in situ *hybridization* in *Arabidopsis thaliana* embryos. Reprinted from Ghosh Dastidar et al. (2016). (E) Immunolocalization of PIN1 (green) in an early‐heart‐stage embryo in *Arabidopsis thaliana*, ecotype *Col‐0*. Adapted from Babić et al. (2025). (F) *Arabidopsis thaliana* embryo at transition stage expressing pDR5rev::3xVENUS‐N7 showing detection of VENUS mRNA (magenta) and VENUS protein (green). Cell wall (white) stained with Renaissance 2200. Scale bar = 20 μm. Adapted from Zhao, Fonseca, et al. (2023). (G) Maximum intensity projections of depth‐coded stacks of actin labeling (*pWOX2:Lifeact:tdTomato*) in a 2‐cell embryo of *Arabidopsis thaliana* . Scale bar = 5 μm. Adapted from Liao and Weijers (2018).

Temperature and other abiotic stresses lead to major changes in expression levels of various genes, requiring the need for protocols to image such transcriptional changes in pollen, ovules, and seeds (Resentini et al. 2023). Different techniques for RNA imaging have been developed, including mRNA in situ hybridization (ISH; Figure 5B; García‐Aguilar et al. 2005) and *single‐molecule* RNA fluorescence in situ hybridization (smRNA‐FISH; Figure 4F; Zhao, Fonseca, et al. 2023). All of them involve the use of antisense RNA probes that hybridize in situ with the RNA of interest. These RNA probes can be visualized using fluorescent or chromogenic approaches. So far, RNA imaging has been performed on both whole mounts and tissue sections.

In whole mounts, mRNA transcripts can be localized at a single‐cell resolution in both sporophytic and gametophytic tissues using in situ hybridization (Figure 5B; García‐Aguilar et al. 2005). The authors applied this method to ovules and early seed development shortly after pollination. RNA in situ hybridization protocols usually use digoxigenin (DIG)‐labeled RNA probes, which are detected in situ by chromogenic or fluorescence‐based methods, depending on the anti‐DIG antibody used for labeling. The fluorescent whole‐mount RNA in situ hybridization (F‐WISH) protocol (Bleckmann and Dresselhaus 2016) is used to visualize mRNA localization on a subcellular level. This method was established in ovules and seeds during early embryo developmental stages, not older than the globular stage (Figure 5A). Whole‐mount protocols cannot be used beyond the globular embryonic stage due to darkening of the endothelium caused by proanthocyanidin accumulation. Solutions would be to use ClearSee Alpha or Fast9 with 60% iohexol for tissue clearing (Attuluri et al. 2022) or to use tissue sections after sample embedding. M2WISH (MicroWave treatment for Whole Mount mRNA In Situ Hybridization) was used to increase the penetration of the RNA probes in thick samples (Chelysheva et al. 2024). This method is compatible with the commonly used cell wall dyes and can be used for the co‐detection of two mRNA probes as double M2WISH. M2WISH was performed on dissected embryos at the heart and subsequent stages (Figure 5C; Chelysheva et al. 2024). Furthermore, they bypassed the problem of proanthocyanidin accumulation by using the pale‐colored seeds of the *tt4* mutant. However, this mutant lacks chalcone synthase, essential for the synthesis of flavonoids (Shirley et al. 1995), and shows reduced heat stress resistance (Yang et al. 2023), which may be problematic for use in phenotyping during abiotic stresses.

To overcome the many limitations of whole‐mount RNA imaging, several studies have used tissue sectioning combined with FISH, notably smRNA‐FISH (Figure 5F). The smFISH technique involves the fluorescent tagging of target transcripts in tissues, allowing for quantification of changes in gene expression levels both spatially and temporally (Duncan et al. 2023; Huang et al. 2019). smFISH of PHYTOCHROME‐INTERACTING FACTOR 4 (PIF4, a key regulator of integration of light and temperature signals) transcripts showed higher expression in tomato anthers under mild low temperature stress (Pan et al. 2021). The advantages of this method are its use in seeds older than post‐globular and the precise quantification of mRNA at single‐cell resolution. Its limitations are the size of the target mRNAs (not smaller than 600 bp) and its low sensitivity for low‐expressed products. The protocol for in situ hybridization of single‐molecule RNA on sections has been adapted for sectioned *Arabidopsis thaliana* embryos (Páldi et al. 2020) or for rice embryos (Yang et al. 2001).

The protocols described above are suitable for larger transcripts. Small RNA molecules, such as miRNA, can be detected by a modified in situ hybridization protocol (Figure 5D; Ghosh Dastidar et al. 2016). It is highly specific, allows for semi‐quantitative assessment of small RNA, and has been tested for ovule and isolated embryos (Ghosh Dastidar et al. 2016). mRNA and miRNA can also be detected simultaneously on a single‐cell level (Wu et al. 2023). However, this protocol has not yet been established for dicots or for embryos and seeds. A limitation of studying only transcriptional changes in response to stress is the often‐observed lack of correlation between transcript and protein abundance. This can be overcome using an approach developed by Zhao, Fonseca, et al. (2023) in which mRNA and proteins are detected simultaneously.

When the use of RNA imaging is not possible, proteins can be localized by immunocytochemistry on whole‐mount and sectioned samples of seeds or other tissues (Figure 5E; Sauer et al. 2006). It requires the availability of specific antibodies against the protein of interest. This method allows for double staining with both antibodies and staining solutions (Sauer et al. 2006) and can be combined with clearing solutions such as ClearSee (Nagaki et al. 2017). If antibodies are not readily available, but the species can be genetically transformed, protein levels can be visualized in reproductive tissues using translational reporter lines where the protein of interest is fused to a fluorescent protein. A growing set of reporter constructs and fluorescence‐based markers has expanded the ability to visualize hormonal gradients, protein localization, and cellular signaling in intact ovules (Cook et al. 2024; De Storme et al. 2016; Pessino et al. 2024; Schubert et al. 2022). These approaches complement morphological imaging by revealing how stress modifies the regulatory networks that govern ovule patterning, embryo development, and overall reproductive success.

At the subcellular level, fluorescent cellular markers developed by Liao and Weijers (2018) provide powerful tools to investigate embryo architecture and connectivity (Figure 5G). Their set of markers allows the visualization of plasma membrane distribution, plasmodesmata, endosomes, the trans‐Golgi network, the tonoplast, nuclear pores, and the cytoskeleton. In addition, they generated markers for apical, basal, central, and peripheral plasma membranes in post‐embryonic tissue, driven by promoters such as *WOX2* (*pWOX2*) and *RIBOSOMAL PROTEIN S5A* (*pRPS5A*). While *pWOX2* is active in early embryonic tissue but decreases after the globular stage, *pRPS5A* remains expressed in meristematic tissues into post‐globular stages (Table 2). Despite their usefulness, these tools face limitations, such as difficulties in imaging cortical microtubule arrays along curved embryo membranes and the lower z‐axis resolution of confocal microscopy. Moreover, their application depends on the ability to generate stable reporter lines, which can be challenging in species recalcitrant to transformation.

### 5.2 Imaging Metabolic Shifts Under Stress

In parallel to changes in the transcriptome, abiotic stresses are often accompanied by a remodeling of the metabolome. Here, we highlight a few imaging approaches that showed changes to the cell wall and inside the seed and specialized metabolites termed flavonols.

The effect of stress on cell wall composition can be analyzed using dyes or immunofluorescent staining of the cell wall sugar polymers. As mentioned above, callose can be stained with aniline blue, β‐glycans, cellulose, and callose with calcofluor (Chebli et al. 2012), acidic polymers and pectic polysaccharides with ruthenium red, and pectin methyl esters with propidium iodide (Chebli et al. 2012). Different antibodies can be used to either determine the methyl esterification grade of pectin (homogalacturonan) molecules using JIM5 for moderate or JIM7 for high methyl esterification (Chebli et al. 2012), or to assess the localization of certain compounds, such as (1 → 4)‐β‐galactan with LM5 and (1 → 5)‐α‐arabinan with LM6 antibodies (Figure 3A; Macquet et al. 2007). However, the penetration of the dye or antibodies through the seed mucilage is a limiting factor and generally incompatible with live‐cell imaging. These challenges can be overcome through embedding and sectioning of seeds in paraffin, plastic, or resin, or by enzymatic digestion of mucilage using pectolytic enzymes (Macquet et al. 2007).

Barley anthers were immune‐stained with antibodies raised against barley (1,3;1,4)‐β‐d‐glucan and LM19 antibodies against homogalacturonan, which showed a change in cell wall composition in response to heat stress (Callens et al. 2023). Although the functional consequences of cell wall remodeling in anthers remain unknown, these results suggest that techniques to analyze the role of cell wall composition in abiotic stress are available. In addition to changes in the anther cell wall composition, heat stress was shown to change callose deposition in growing pollen tubes (Ouonkap et al. 2024).

Changes of metabolites like aromatics, carbohydrates, as well as lipids and water inside plant tissues under stress can be determined using NMR‐based chemical shift imaging (CSI) (Melkus et al. 2009; Neuberger et al. 2008; Rumpel and Pope 1992). For NMR‐based CSI, protons of atoms are showing distinct peaks, which are later converted into images, along the *x*‐axis of the NMR spectra, enabling the determination of a molecule's structure (Neuberger et al. 2008). Using this method, Neuberger et al. (2008) detected lipid storage in barley and soybean seeds and were able to determine tissue‐specific lipid localization and gradient inside the seed (Figure 3E). Importantly, they showed that NMR‐based CSI is not dependent on the lipid content of specific anatomical structures: it was successfully applied both to barley seeds with low lipid levels and to soybean seeds with high lipid content and strong lipid compartmentalization. This approach also has the potential to monitor stress responses in the endosperm; for example, Melkus et al. (2009) tested it for hypoxia and anoxia, but they did not find any differences in the levels of sucrose, alanine, and glutamine. Key advantages of this method are that the sample is not damaged during sample preparation, and CSI allows for long repetition time and robust metabolite quantification with minimal post‐processing. However, the method has limitations, such as the authors not obtaining any spatial resolution of the layers in the endosperm cytoplasm, as NMR‐based CSI is not well suited for detecting low‐concentration metabolites (lower than 10 mM by a voxel volume of 0.56 μL) (Ratcliffe and Shachar‐Hill 2001). Furthermore, it is time‐consuming, as calibration with gas chromatography is required to convert the NMR signal intensity into actual metabolite content. In addition, MRI has also been used to detect changes in the distribution of metabolites in the tissues of *Brassica napus* seeds and the endosperm of *Pisum sativum* (Borisjuk et al. 2012; Melkus et al. 2009; Rolletschek et al. 2021).

Profiling of specialized metabolites, such as flavonols, can also be used as a measure of stress response due to the protective role of flavonols during abiotic stress through their ROS scavenging capacity (Daryanavard et al. 2023). Flavonols can be selectively stained with the fluorescent dye diphenylboric acid 2‐aminoethylester (DPBA) (Figure 1B; Nguyen 2020). Pollen tubes stained with DPBA of a tomato flavonol overexpression line showed higher fluorescence intensity along with enhanced pollen germination and tube growth under heat stress (Postiglione et al. 2024). Moreover, DPBA‐stained pollen grains and tubes of a tomato flavonol‐deficient mutant showed lower intensity and high susceptibility to heat stress in terms of pollen viability, germination, and tube growth (Muhlemann et al. 2018). Flavonol imaging can be simultaneously performed with certain ROS‐specific stains for parallel analysis of flavonol and ROS levels in response to stress.

Although water is not a metabolite per se, it plays a central role in metabolism and physiology. Importantly, the cell's water content and water potential fluctuate widely as a function of diverse abiotic stresses (Feng et al. 2016). A recent study developed a minimally disruptive hydrogel nanoreporter called AquaDust to visualize and measure leaf water potential (Jain et al. 2021). This tool still has to be implemented in reproductive tissues.

## 6 Current Limitations in Imaging Plant Reproductive Development Under Stress

Despite substantial progress in imaging approaches, plant reproductive tissues remain technically challenging to visualize across the entire developmental trajectory, especially under abiotic stress. During pollen development and tube growth, autofluorescence of pollen grains interferes with fluorescent staining, and most studies rely on endpoint assays with dyes such as CM‐H_2_DCFDA, DHE, or aniline blue, which capture only static snapshots of ROS or callose deposition.

Imaging the female gametophyte and subsequent fertilization stages presents additional barriers. Deep‐tissue visualization requires tissue clearing and staining, limiting the feasibility of live‐cell imaging. Ca^2+^ dynamics remain minimally explored in ovules, and ROS imaging is primarily limited to endpoint histochemical stains, with no real‐time monitoring using genetically encoded reporter constructs routinely applied in these tissues. High‐resolution 3D imaging is further constrained by optical complexity and autofluorescence.

Challenges intensify during post‐fertilization seed development, where tannin and phenolic accumulation in the seed coat reduces tissue transparency, making imaging beyond the globular embryo stage difficult even with confocal or cleared‐tissue approaches. Research on stress responses in the embryo, endosperm, and seed coat is extremely scarce, especially for live‐cell imaging. Monitoring ROS and Ca^2+^ in these tissues is currently dependent on chemical dyes, which are prone to non‐quantitative, irreversible, and not suitable for dynamic time‐course analysis. While genetically encoded sensors such as HyPer (H_2_O_2_) and GECIs (Ca^2+^) could provide real‐time insights, their use is hampered by the need for stable transformation, which remains a major bottleneck in recalcitrant crops.

Single‐molecule RNA‐FISH has enabled high‐resolution mapping of transcriptional responses, but it is low‐throughput, requires specialized microscopy, and currently lacks robust protocols for plant reproductive tissues, most of which are adapted from mammalian systems. Moreover, smFISH has not been broadly applied to stress‐response studies in different crops or tissue types, and protocol optimization and publication remain an unmet need.

Overall, current imaging techniques are low‐throughput, invasive, and largely limited to static observations, leaving significant knowledge gaps in tissue‐specific, dynamic stress responses during plant reproduction. Future advances will require non‐invasive, high‐resolution, and genetically encoded imaging platforms to enable real‐time, multi‐tissue monitoring of stress signals from pollen to seed.

Summary of current limitations in reproductive tissue imaging under stress:

- *Pollen development*: Autofluorescence complicates staining; ROS imaging is mostly endpoint; smRNA‐FISH is low‐throughput and under‐optimized for plants.
- *Ovule development*: Limited live‐cell imaging; Ca^2+^ minimally studied; ROS mostly via static chromogenic and fluorescent stains.
- *Fertilization and early seed development*: Tannin accumulation hinders imaging beyond the globular stage; deep‐tissue autofluorescence limits confocal and cleared‐tissue imaging.
- *Post‐fertilization* (embryo, endosperm, seed coat): Stress‐response imaging is scarce; ROS and Ca^2+^ detection relies on dyes; real‐time biosensors are underutilized due to transformation bottlenecks.
- *Cross‐cutting limitations*: Low throughput, advanced microscopy expertise required, few plant‐specific protocols, lack of dynamic, tissue‐specific reporters, lack of genetically encoded sensors expressed in (crop) reproductive tissues.

Future progress depends on non‐invasive, high‐resolution, genetically encoded imaging platforms enabling real‐time, multi‐tissue monitoring from pollen to seed.

## Author Contributions

M.A.M., H.S.R., F.R., and J.K.M. outlined the content of the manuscript. H.D., T.P., M.C.P., and B.M.O.M. drafted the manuscript and prepared the figures. H.S.R., F.R., and J.K.M. have revised the manuscript and figures. All the authors have read and approved the final manuscript.

## Funding

This article has benefited from networking opportunities provided by the COST Action RECROP (CA22157), supported by COST (European Cooperation in Science and Technology). Joëlle K. Mühlemann and Hana Daryanavard were supported by a KU Leuven startup grant (STG/21/019). Teresa Paraiso and Hélène S. Robert were supported by the project TowArds Next GENeration Crops (TANGENC), reg. No. CZ.02.01.01/00/22_008/0004581, of the Programme Johannes Amos Comenius (OP JAK) from the Ministry of Education, Youth and Sports of the Czech Republic (MEYS). Francesca Resentini was supported by the University of Milan with the Linea 3—Bando Straordinario per Progetti Interdipartimentali (G45F21003110005), while Bianca Maria Orlando Marchesano was supported by the Agritech National Research Center, funded by the European Union NextGenerationEU (Piano Nazionale di Ripresa e Resilienza (PNRR)—Missione 4, Componente 2, Investimento 1.4—D.D. 1032 17/06/2022, CN00000022). Marta Adelina Mendes was supported by the Italian Ministry of University and Research (2022) PRIN—Progetti di Ricerca di Rilevante Interesse Nazionale (Grant No. 20229K8ZWF).

## Disclosure

Minor parts of the text were improved using AI, notably for grammar and sentence structure. All the modified text was proofread and adjusted by the authors.

## Conflicts of Interest

The authors declare no conflicts of interest.

## Data Availability

No new data were generated or analysed in this study.
